# Correlation of Early Nutritional Supply and Development of Bronchopulmonary Dysplasia in Preterm Infants <1,000 g

**DOI:** 10.3389/fped.2021.741365

**Published:** 2021-10-07

**Authors:** Theresa Thiess, Tina Lauer, Annika Woesler, Janine Neusius, Sandro Stehle, Klaus-Peter Zimmer, Gunter Peter Eckert, Harald Ehrhardt

**Affiliations:** ^1^Department of General Pediatrics and Neonatology, Justus-Liebig-University, Universities of Gießen and Marburg Lung Center (UGMLC), German Center for Lung Research (DZL), Gießen, Germany; ^2^Department of Nutritional Sciences, Institute for Nutrition in Prevention and Therapy, Justus-Liebig-University, Gießen, Germany

**Keywords:** chronic lung disease (CLD), bronchopulmonary dysplasia (BPD), preterm infant, nutrition, caloric intake, enteral supply, feeding intolerance, low birth weight infant

## Abstract

**Background:** Bronchopulmonary dysplasia (BPD) has multifactorial origins and is characterized by distorted physiological lung development. The impact of nutrition on the incidence of BPD is less studied so far.

**Methods:** A retrospective single center analysis was performed on n = 207 preterm infants <1,000 g and <32 weeks of gestation without severe gastrointestinal complications to assess the impact of variations in nutritional supply during the first 2 weeks of life on the pulmonary outcome. Infants were grouped into no/mild and moderate/severe BPD to separate minor and major limitations in lung function.

**Results:** After risk adjustment for gestational age, birth weight, sex, multiples, and antenatal steroids, a reduced total caloric intake and carbohydrate supply as the dominant energy source during the first 2 weeks of life prevailed statistically significant in infants developing moderate/severe BPD (*p* < 0.05). Enteral nutritional supply was increased at a slower rate with prolonged need for parenteral nutrition in the moderate/severe BPD group while breast milk provision and objective criteria of feeding intolerance were equally distributed in both groups.

**Conclusion:** Early high caloric intake is correlated with a better pulmonary outcome in preterm infants <1,000 g. Our results are in line with the known strong impact of nutrient supply on somatic growth and psychomotor development. Our data encourage paying special attention to further decipher the ideal nutritional requirements for unrestricted lung development and promoting progressive enteral nutrition in the absence of objective criteria of feeding intolerance.

## Introduction

Bronchopulmonary dysplasia (BPD) is a chronic lung disease of the preterm infant characterized by distorted physiological lung growth (1, 2). The restrictions in pulmonary function are associated with long-term respiratory illnesses. Furthermore, BPD predisposes for disturbance of further somatic growth and psychomotor development (3, 4).

Inflammatory processes in the immature lung caused by mechanical ventilation and oxygen toxicity are the main contributors to the disease (1). Pre- and postnatal infections and alterations in the microbiome in the lung contribute to the multifactorial etiology (5). Intrauterine growth restriction is one further key risk for BPD identified in many large cohort studies, whereas the impact of differences in postnatal nutritional intake and fluid supply just recently opened new therapeutic options to reduce BPD. Sparse retrospective studies evaluated the impact of early nutritional supply on the pulmonary outcome in more detail: A lower enteral intake of carbohydrates and lipids during the first 14 days of life and a lower total supply of calories and protein during the first 4 weeks of life predisposed preterm infants below 28 weeks of gestation for BPD and retinopathy of prematurity (6–10). Two publications documented the particular benefit of rapid advances in enteral nutrition to reduce the risk of BPD (11, 12). The recent meta-analysis on the benefits of an early rapid increase in enteral nutrition confirms the safety of such an approach (13). Early full enteral feed and the provision of breast milk are suited to promote growth and to reduce the incidence of BPD (14, 15). Furthermore, vitamin A is the best-studied supplement to reduce the risk of BPD although its effect is moderate (16).

This gap of knowledge in the context of BPD pathogenesis is surprising, as the short-term benefits of high caloric nutritional supply on somatic growth and psychomotor development are reasonably well-established. Therefore, current nutritional guidelines and recommendations are mainly derived from the scientific data obtained for motor function measures and cognitive outcomes. The recommended high protein intake and the proper distribution of carbohydrates, proteins, and lipids reduce the risk for lifelong sequelae following preterm birth. The evaluation of lipid composition and adequate supply of micronutrients demonstrates the opportunity to further optimize nutritional supply and the effect size on these outcome measures (17, 18). On the other hand, aggressive nutritional supply in the neonatal intensive care unit (NICU) predisposes for later metabolic disorders in the preterm infant, including atherogenic lipid profiles and higher arterial blood pressures (19, 20). Therefore, known benefits and risks have to be weighed up carefully.

In our retrospective single center cohort study, we aimed to correlate the nutritional supply within the first 2 weeks of life and the occurrence of moderate/severe BPD in more than 200 preterm infants with a birth weight <1,000 g. Datasets were analyzed before and after risk adjustment for important clinical confounders. Further detailing of the nutritional supply was intended to specify the contribution of macronutrients and supplements with known impact on the preterm infant's outcomes.

## Patients and Methods

### Patient Cohort, Exclusion Criteria, and Ethics Vote

Based on the results from a population-based cohort study in 10 European regions including our region of Hesse, we focused on infants at highest risk for moderate/severe BPD with a birth weight <1,000 g and gestational age <32 + 0 weeks (21). Nutritional datasets were available for all infants born since 2006 within the retrospective NuPre (Nutrition Preterm) cohort study at our tertiary perinatal center (Justus-Liebig-University Gießen, Germany), and all infants born between 2006 and 2017 were included in the analyses. An *a priori* power calculation was not performed. The aim was to correlate the nutritional supply and the pulmonary outcome. From *n* = 295 available patients, *n* = 88 preterm infants were excluded due to death before 36 weeks of gestation (*n* = 56), severe congenital malformation of the lung, heart, brain, gut, or kidneys (*n* = 9), presence of severe gastrointestinal complications of necrotizing enterocolitis (NEC, *n* = 6) or focal intestinal perforation (*n* = 6), infection with cytomegalovirus (*n* = 1), and severe aspiration pneumonia (*n* = 1). Furthermore (*n* = 9) preterms had to be excluded due to the transfer to another NICU before 36 weeks (*n* = 6), outborn status (*n* = 2), or missing data (*n* = 1). We decided to exclude deaths that were mostly related to death in the delivery room (*n* = 10), death during unrestricted NICU therapy independent of the pulmonary status (*n* = 20), decision to cessation of NICU therapy independent of the pulmonary status (*n* = 18), and severe congenital malformations (*n* = 6), while only two deaths were due to respiratory failure during intensive care therapy (2/56, 3.6%). Furthermore, infants with NEC were excluded due to the high impact of NEC pathology on BPD.

The study was conducted following the rules of the Declaration of Helsinki of 1975, revised in 2013, and was approved by the ethics committee of the Justus-Liebig-University Gießen (AZ 98/14).

### Data Acquisition and Parameter Definition

All parameters were obtained from the electronic data management systems and the paper file records. Data were entered into a SPSS Statistics databank version 23.0.0.3 for Microsoft Windows (IBM, Armonk, NY). Baseline maternal and infant parameters included gestational age, birth weight, sex, birth as singleton, or multiples, and antenatal steroid application. Percentiles for weight, length, and head circumference were derived from the German perinatal registry (22). Small for gestational age (SGA) was defined as birthweight <10th percentile. Antenatal steroids were categorized as no completed course (<48 h), delivery within 7 days after the last application and provision of antenatal steroids more than 7 days before birth. Enteral and parenteral nutritional supply was recorded on the day of birth (day 1) and thereafter on days 3, 5, 7, 10, and 14 of life. The average nutritional supply was chosen as readout to respect the large variations in nutritional advances within the observation period. It was calculated as the mean of the collected time points. The supply was recorded for all nutrients, total caloric, and fluid intake. Total fluid intake comprises parenteral and enteral nutritional supply and all continuous infusions applied with a duration >1 h but no bolus medication. Duration of parenteral nutrition was documented in days. Enteral nutrition was separated by the volume and type of formula, milk enrichment with fortifiers, and the provision of breast milk. The composition of breast milk was calculated based on the scientific data and the expected changes in composition over time (23–27). Changes in formula composition during the study period were supplied by all manufacturers and were integrated into the calculations starting in the first month after the change. Signs for feeding intolerances such as distended abdomen, vomiting, biliary, and hemorrhagic remains in the stomach, hemorrhagic feces, and reduced or paused enteral feed supply were collected from the clinical documentation system, including medical and nursing notes, and aggregated for analyses. Decrease in enteral feed comprises any reduction in quantity compared with the reached level while pause of feeding comprises any cessation of enteral nutrition independent of the duration. Due to the retrospective nature of our study, more precise separations of feeding intolerance were not feasible.

Severity of BPD was determined as no, mild, moderate, or severe BPD according to the NICHD consensus definition that is most widely used within the literature (28). Support with highflow nasal cannula was assessed as continuous positive airway pressure (CPAP) whenever a level of 3-cm H_2_O or higher was attained and the fraction of oxygen provided by nasal cannula was determined as published (29, 30).

### Statistical Analysis

As previously, the statistical analysis was directed toward the separation of infants without/with mild BPD from infants with moderate/severe BPD as the latter infants have relevant limitations in lung function (31). The severity of BPD is highly impacted by gestational age, birth weight, sex, birth as multiple, and the optimized application of antenatal steroids (32). Data were analyzed before and after risk adjustment for these baseline confounders using partial correlation.

Cohort characteristics were compared using Mann–Whitney U and Chi-squared test or Spearman's correlation coefficient. Statistical analysis was performed using partial correlation and IBM SPSS Statistics Version 23.0.0.3 for Microsoft Windows. Statistical significance was accepted at *p* < 0.05.

## Results

Overall, *n* = 207 infants were available for statistical analysis, of which *n* = 50 infants (24.2%) did not fulfill any BPD criterion according to the NICHD consensus definition. The majority of *n* = 94 infants (45.4 %) was assigned to mild BPD which are known to have a lung function close to preterm infants not fulfilling the criteria of BPD (33). The *n* = 63 infants with moderate or severe BPD and considerably restricted pulmonary outcome constituted about one third (30.4%) of the total cohort (Table 1). Within the study period of more than 10 years, the incidence of BPD did not differ significantly between the years (*p* = 0.578) in-line with the published literature (34).

**Table 1:** Patient cohort characteristics separated for the severity of bronchopulmonary dysplasia (BPD).

	**No/mild BPD** ***n* = 144**	**Moderate/severe BPD** ***n* = 63**	***p*-value**
Gestational age (weeks)	27 + 2 (25 + 6; 28 + 5)	25 + 0 (24 + 2; 26 + 2)	**<0.0001**
Proportion of infants <28 weeks of gestation	94 (65.3)	59 (93.7)	**0.005**
Birth weight (g)	880 (740; 960)	680 (530; 790)	**<0.0001**
Proportion of infants <750 g	42 (29.2)	42 (66.7)	0.323
Small for gestational age	31 (21.5)	19 (30.2)	0.182
Male sex	51 (35.4)	37 (58.7)	0.002
Multiple birth	42 (29.2)	22 (34.9)	0.412
No antenatal steroids	24 (16.7)	9 (14.3)	
Antenatal steroids ≤ 7 days	67 (46.5)	37 (58.7)	
Antenatal steroids >7 days	53 (36.8)	17 (27.0)	0.924
Surfactant therapy	81 (56.3)	54 (85.7)	**<0.0001**
Invasive mechanical ventilation (days)	2 (0; 29)	21 (0; 187)	**<0.0001**
Ventilatory support (days)	29 (0; 77)	62 (13; 165)	**0.001**

*Data are presented as n (%) or median and interquartile range (Q1; Q3), if not indicated differently. p < 0.05, Mann–Whitney-U or Chi-squared test. The statistically significant results are indicated by bold print. Ventilatory support comprises the total duration of all modes of non-invasive ventilation with provision of positive end expiratory pressure of 3-cm H_2_O or higher*.

The severity of BPD was significantly affected by the gestational age, birth weight, and male sex while further risk factors such as being small for gestational age, multiple births, and timing of antenatal steroid application did not reach statistical significance (Table 1). In line with the published literature, infants from the moderate/severe BPD group depended longer on invasive mechanical ventilation as well as non-invasive ventilatory support, and required surfactant therapy more frequently (Table 1) (32).

### Reduced Caloric Intake During the First 2 Weeks of Life in Infants With Moderate/Severe Bronchopulmonary Dysplasia

**Figure 1:**
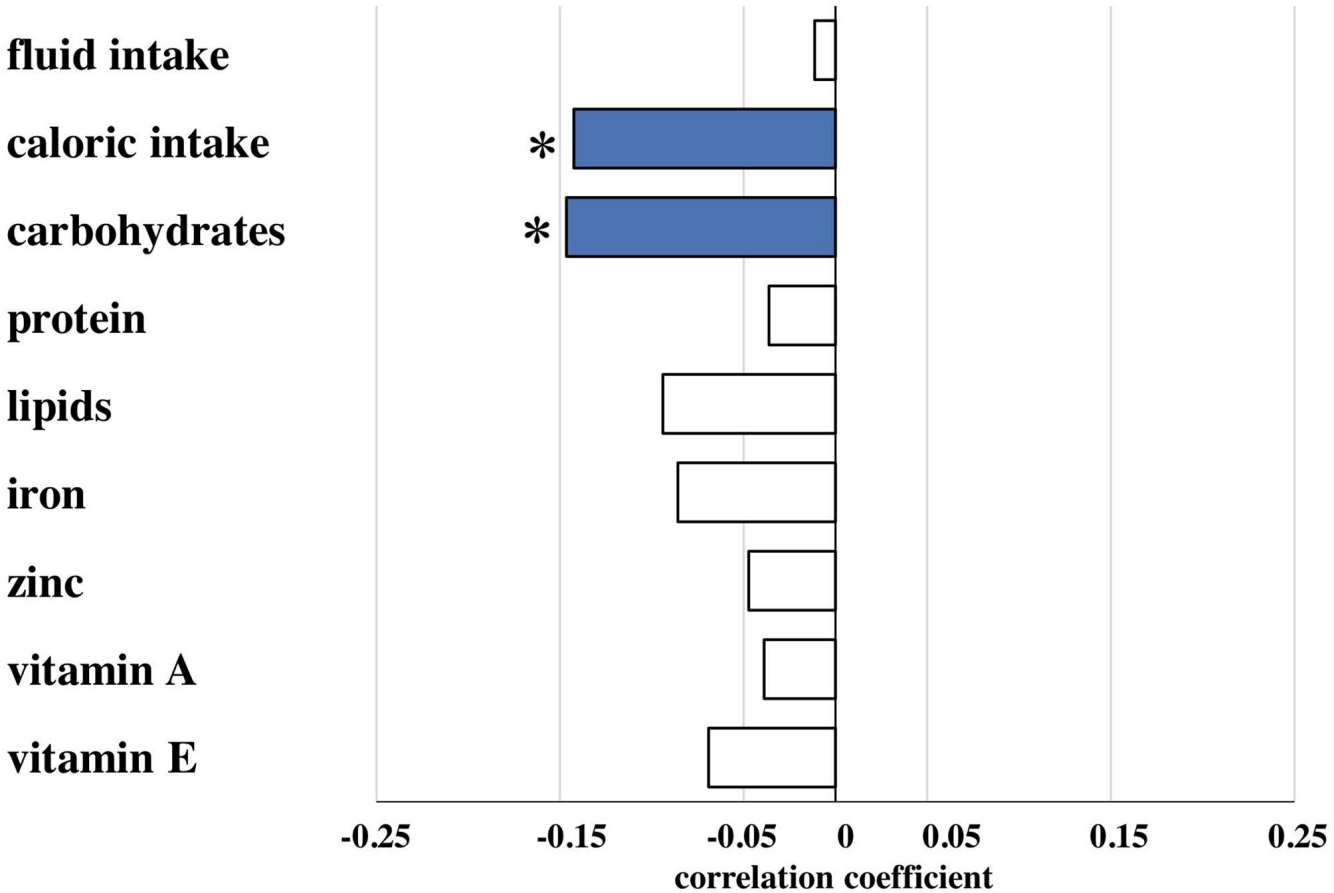
Partial correlation of nutritional components during the first 2 weeks of life and bronchopulmonary dysplasia (BPD). Correlation coefficients are provided for nutritional components and presence of moderate/severe BPD for total fluid (ml/kg), total caloric (kcal/kg), carbohydrate (g/kg), protein (g/kg), lipid (g/kg), iron (mg/kg), zinc (mg/kg), vitamin A (mg/kg) and vitamin E (mg/kg) intake calculated from the total (enteral plus parenteral) nutritional supply during the first 14 days of life as described in patients and methods. Risk adjustment was performed for gestational age, birth weight, sex, multiple birth, and antenatal steroid exposure. Nutritional data are presented in detail in Table 2. *Spearman's correlation coefficient with a significance of *p* < 0.05 (blue bars).

**Figure 2:**
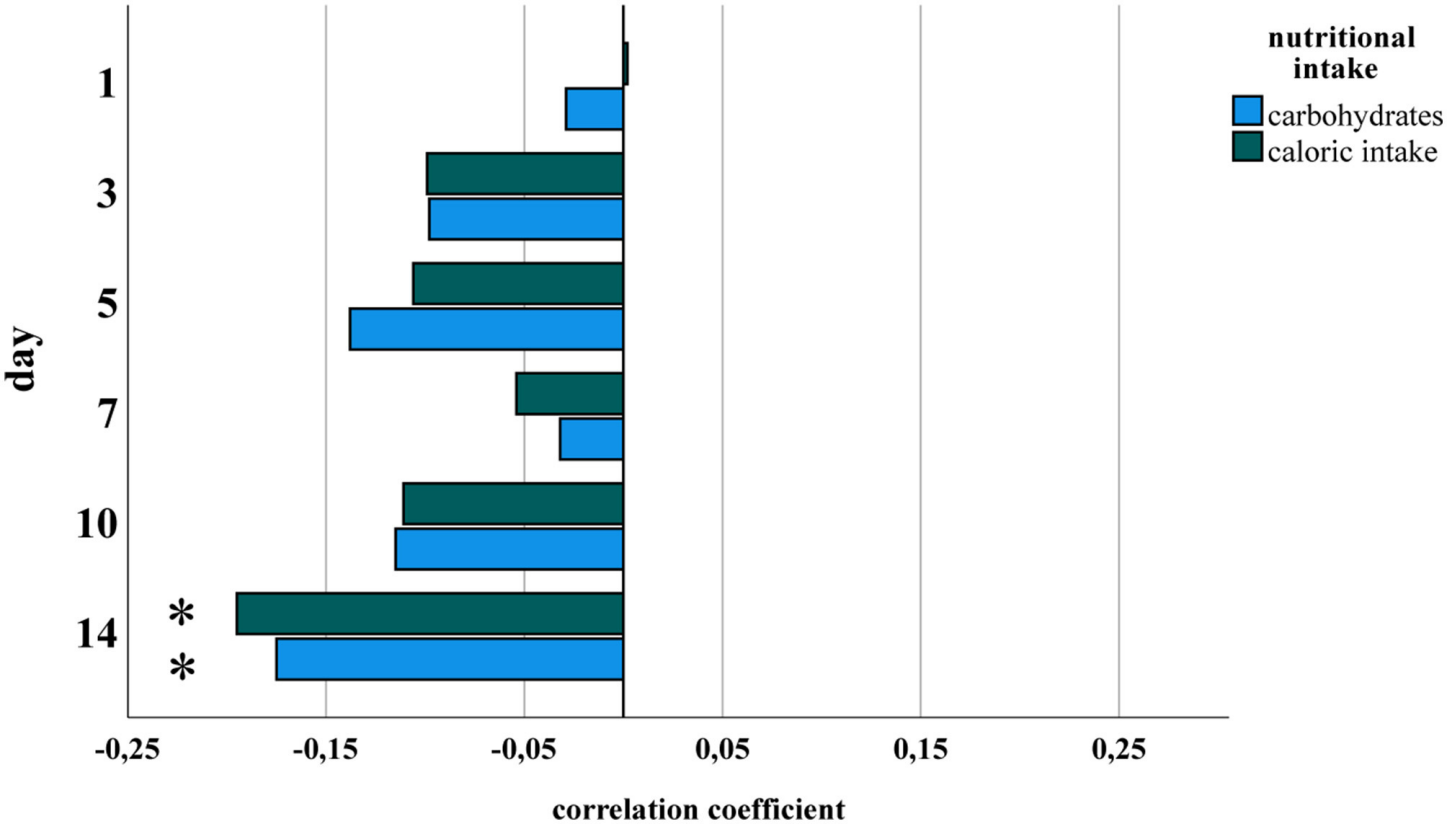
Partial correlation of nutritional components and BPD for each study point. Datasets from Figure 1 were analyzed separately for days 1, 3, 5, 7, 10, and 14 of life for total caloric intake and carbohydrate supply. Statistical analysis was performed as in Figure 1. *Spearman's correlation coefficient with a significance of p < 0.05.

Amounts of total fluid intake, total caloric supply, and carbohydrate and lipid supply were significantly higher prior to risk adjustment in infants belonging to the no/mild BPD group. As an exception, protein intake did not differ between both groups. After risk adjustment reduced total caloric intake and supply with carbohydrates correlated significantly with moderate/severe BPD (Table 2; Figure 1). Additional risk adjustment for SGA did not alter the results (Table 3). A separate investigation of *n* = 153 infants below 28 weeks of gestation revealed an even more pronounced difference (Table 4), while in the subgroup of infants with a birth weight <750 g (*n* = 84), the analyses did not reach statistical significance (Table 5). Detailed investigation revealed a trend toward lower total caloric intake and carbohydrate supply at each investigation time point except for day 1. Statistical significance was reached on day 14 (Figure 2).

**Table 2:** Average daily total nutritional intake during the first 14 days.

	**No/mild BPD** ***n* = 144**	**Moderate/severe BPD** ***n* = 63**	**Before risk adjustment**		**After risk adjustment^a^**	
			**rho**	***p*-value**	**rho**	***p*-value**
Total fluid intake (ml/kg)	129.73 (116.82; 144.08)	108.81 (91.19; 129.20)	**−0.313**	**<0.0001**	−0.011	0.873
Total caloric intake (kcal/kg)	94.27 (86.53; 100.32)	85.92 (76.22; 94.96)	**−0.269**	**<0.0001**	**−0.142**	**0.044**
Carbohydrates (g/kg)	11.99 (10.88; 12.98)	10.83 (9.59; 11.58)	**−0.341**	**<0.0001**	**−0.146**	**0.038**
Protein (g/kg)	4.26 (3.15; 5.30)	4.04 (3.23; 5.50)	−0.001	0.994	−0.036	0.610
Lipids (g/kg)	3.38 (2.85; 3.84)	3.04 (2.54; 3.60)	**−0.185**	**0.008**	−0.094	0.184
Iron (mg/kg)	1.07 (0.44; 1.74)	0.60 (0.22; 1.20)	**−0.249**	**<0.0001**	−0.086	0.226
Zinc (mg/kg)	0.89 (0.70; 1.03)	0.80 (0.60; 0.95)	**−0.144**	**0.038**	−0.047	0.506
Vitamin A (mg/kg)	0.41 (0.16; 0.68)	0.24 (0.13; 0.67)	−0.064	0.360	−0.039	0.584
Vitamin E (mg/kg)	2.36 (1.89; 2.9)	2.03 (1.55; 2.54)	**−0.182**	**0.009**	−0.069	0.330

*Data are presented as median and interquartile range (Q1; Q3). p < 0.05, Spearman's correlation coefficient. The statistically significant results are indicated by bold print*.

a *After risk adjustment for gestational age, birth weight, sex, multiple birth, and antenatal corticosteroid exposure*.

**Table 3:** Average daily total nutritional intake during the first 14 days with additional risk adjustment for small for gestational age (SGA).

	**No/mild BPD** ***n* = 144**	**Moderate/severe BPD** ***n* = 63**	**Before risk adjustment**		**After risk adjustment^a^**	
			**rho**	***p*-value**	**rho**	***p*-value**
Total fluid intake (ml/kg)	129.73 (116.82; 144.08)	108.81 (91.19; 129.20)	**−0.313**	**<0.0001**	−0.007	0.927
Total caloric intake (kcal/kg)	94.27 (86.53; 100.32)	85.92 (76.22; 94.96)	**−0.269**	**<0.0001**	**−0.140**	**0.048**
Carbohydrates (g/kg)	11.99 (10.88; 12.98)	10.83 (9.59; 11.58)	**−0.341**	**<0.0001**	**−0.146**	**0.039**
Protein (g/kg)	4.26 (3.15; 5.30)	4.04 (3.23; 5.50)	−0.001	0.994	−0.027	0.700
Lipids (g/kg)	3.38 (2.85; 3.84)	3.04 (2.54; 3.60)	**−0.185**	**0.008**	−0.097	0.173
Iron (mg/kg)	1.07 (0.44; 1.74)	0.60 (0.22; 1.20)	**−0.249**	**<0.0001**	−0.094	0.186
Zinc (mg/kg)	0.89 (0.70; 1.03)	0.80 (0.60; 0.95)	**−0.144**	**0.038**	−0.049	0.487
Vitamin A (mg/kg)	0.41 (0.16; 0.68)	0.24 (0.13; 0.67)	−0.064	0.360	−0.040	0.570
Vitamin E (mg/kg)	2.36 (1.89; 2.9)	2.03 (1.55; 2.54)	**−0.182**	**0.009**	−0.074	0.295

*Data are presented as median and interquartile range (Q1; Q3). p < 0.05, Spearman's correlation coefficient. The statistically significant results are indicated by bold print*.

a *After risk adjustment for gestational age, birth weight, sex, multiple birth, antenatal corticosteroid exposure, and SGA*.

**Table 4:** Average daily total intake of infants <28 weeks of gestation during the first 14 days.

	**No/mild BPD** ***n* = 94**	**Moderate/severe BPD** ***n* = 59**	**Before risk adjustment**		**After risk adjustment^a^**	
			**rho**	***p*-value**	**rho**	***p*-value**
Total fluid intake (ml/kg)	72.82 (0.00; 140.10)	58.84 (26.49; 122.77)	**−0.342**	**<0.0001**	0.000	0.999
Total caloric intake (kcal/kg)	39.40 (0.00; 80.33)	40.73 (22.31; 77.06)	**−0.286**	**<0.0001**	**−0.169**	**0.041**
Carbohydrates (g/kg)	7.15 (0.00; 12.36)	7.43 (4.67; 13.76)	**−0.375**	**0.008**	**−0.210**	**0.011**
Protein (g/kg)	1.61 (0.00; 3.41)	1.56 (0.77; 2.83)	−0.038	0.641	−0.037	0.658
Lipids (g/kg)	0.38 (0.00; 2.43)	0.31 (0.00; 3.20)	−0.148	0.068	−0.063	0.447
Iron (mg/kg)	0.15 (0.00; 0.29)	0.13 (0.00; 0.37)	**−0.201**	**0.013**	−0.070	0.402
Zinc (mg/kg)	0.11 (0.00; 0.26)	0.09 (0.00; 0.25)	−0.130	0.110	−0.057	0.496
Vitamin A (mg/kg)	0.03 (0.00; 0.06)	0.03 (0.00; 0.17)	−0.067	0.409	−0.060	0.474
Vitamin E (mg/kg)	0.31 (0.00; 0.60)	0.25 (0.00; 1.55)	−0.152	0.060	−0.066	0.428

*Data are presented as median and interquartile range (Q1; Q3). p < 0.05, Spearman's correlation coefficient. The statistically significant results are indicated by bold print*.

a *After risk adjustment for gestational age, birth weight, sex, multiple birth, and antenatal corticosteroid exposure*.

**Table 5:** Average daily total intake of infants with a birth weight <750 g during the first 14 days.

	**No/mild BPD** ***n* = 42**	**Moderate/severe BPD** ***n* = 42**	**Before risk adjustment**		**After risk adjustment^a^**	
			**rho**	***p*-value**	**rho**	***p*-value**
Total fluid intake (ml/kg)	64.50 (36.04; 104.00)	56.16 (26.49; 93.00)	**−0.314**	**0.004**	−0.118	0.305
Total caloric intake (kcal/kg)	40.00 (26.62; 70.85)	41.33 (23.29; 77.06)	**−0.319**	**0.003**	−0.113	0.323
Carbohydrates (g/kg)	7.33 (4.56; 11.23)	7.43 (4.88; 12.08)	**−0.344**	**0.001**	−0.098	0.395
Protein (g/kg)	1.64 (0.91; 2.99)	1.57 (0.80; 2,83)	−0.047	0.670	0.047	0.681
Lipids (g/kg)	0.38 (0.00; 2.43)	0.35 (0.00; 3.20)	**−0.248**	**0.023**	−0.105	0.360
Iron (mg/kg)	0.17 (0.00; 0.29)	0.15 (0.00; 0.37)	−0.195	0.075	−0.075	0.517
Zinc (mg/kg)	0.11 (0.00; 0.19)	0.10 (0.00; 0.25)	−0.153	0.164	−0.079	0.491
Vitamin A (mg/kg)	0.03 (0.00; 0.06)	0.03 (0.00; 0.17)	−0.038	0.729	−0.018	0.878
Vitamin E (mg/kg)	0.30 (0.00; 0.59)	0.28 (0.00; 1.55)	−0.174	0.114	−0.110	0.336

*Data are presented as median and interquartile range (Q1; Q3). p < 0.05, Spearman's correlation coefficient. The statistically significant results are indicated by bold print*.

a *After risk adjustment for gestational age, birth weight, sex, multiple birth, and antenatal corticosteroid exposure*.

To exclude that the observed effects are due to differences in the provision of vitamins and antioxidants, we evaluated the supplementation with iron, zinc, vitamin A, and vitamin E. Only vitamin A intake did not differ between both groups before risk adjustment. In contrast to the nutritional intake, no statistically significant differences in those supplements were detected after risk adjustment (Tables 2, 3; Figure 1).

### Reduced Enteral Nutrition Accountable for Nutritional Deficits in Infants With Moderate/Severe Bronchopulmonary Dysplasia in the First 2 Weeks of Life

Enteral nutritional supply was started in all infants within the first hours of life. Separate consideration of the enteral nutrition supply revealed a trend toward statistically non-significant increases in enteral supply (50.44 ml/kg/day in the no/mild BPD group vs. 20.74 ml/kg/day in the moderate/severe BPD group). In line, the enteral intake of calories, macronutrients and supplements tended to be higher in the no/mild BPD group, however without statistical significance (Table 6). The duration of parenteral nutrition (15 vs. 21 days) was significantly shorter in infants with no/mild BPD (Table 7).

**Table 6:** Average daily enteral nutritional intake during the first 14 days.

	**No/mild BPD** ***n* = 144**	**Moderate/severe BPD** ***n* = 63**	**Before risk adjustment**		**After risk adjustment^a^**	
			**rho**	***p*-value**	**rho**	***p*-value**
Total fluid intake (ml/kg)	50.44 (31.10; 69.50)	20.74 (13.91; 38.19)	**−0.459**	**<0.0001**	−0.092	0.195
Total caloric intake (kcal/kg)	50.30 (35.79; 61.32)	36.41 (22.93; 53.50)	**−0.255**	**<0.0001**	−0.073	0.306
Carbohydrates (g/kg)	5.65 (3.89; 6.91)	4.18 (2.28; 5.92)	**−0.248**	**<0.0001**	−0.067	0.347
Protein (g/kg)	2.09 (1.42; 3.16)	1.66 (0.76; 2.82)	−0.147	**<0.0001**	−0.071	0.317
Lipids (g/kg)	2.38 (1.80; 2.89)	1.88 (1.15; 2.53)	**−0.259**	**<0.0001**	−0.072	0.310
Iron (mg/kg)	1.07 (0.44; 1.74)	0.60 (0.22; 1.20)	**−0.249**	**<0.0001**	−0.086	0.226
Zinc (mg/kg)	0.89 (0.70; 1.03)	0.80 (0.60; 0.95)	**−0.144**	**0.038**	−0.047	0.506
Vitamin A (mg/kg)	0.34 (0.10; 0.63)	0.16 (0.06; 0.60)	−0.103	0.141	−0.045	0.528
Vitamin E (mg/kg)	1.80 (1.29; 1.38)	1.37 (0.85; 2.05)	**−0.226**	**0.001**	−0.075	0.289

*Data are presented as median and interquartile range (Q1; Q3). p < 0.05, Spearman's correlation coefficient. The statistically significant results are indicated by bold print*.

a *After risk adjustment for gestational age, birth weight, sex, multiple birth, and antenatal corticosteroid exposure*.

**Table 7:** Separation of clinical reasons for delayed increments in enteral nutrition during the first 14 days of life.

	**No/mild BPD** ***n* = 144**	**Moderate/severe BPD** ***n* = 63**	***p*-value**
Feeding intolerance day 1–14	141 (97.9)	63 (100.0)	0.248
Feeding intolerance day 1	32 (22.2)	13 (20.6)	0.799
Reduced enteral nutrition, number of cases	53 (36.8)	24 (38.1)	0.860
Paused enteral nutrition, number of cases	5 (3.5)	3 (4.8)	0.658
Enteral nutrition reduced, total number of events	65 (7.5)	37 (9.8)	0.183
Enteral nutrition paused, total number of events	119 (13.8)	79 (20.9)	**0.002**
Breast milk supply, number of cases	71 (49.3)	30 (47.6)	0.823
Milk fortification, number of cases	104 (72.2)	45 (71.4)	0.232
Duration parenteral nutrition (days)	15 (13; 18)	21 (15; 18)	**0.002**

*Data are presented as n (%) besides duration of parenteral nutrition [median and interquartile range (Q1; Q3)]. p < 0.05 (bold print), Mann–Whitney U or Chi-squared test. Reduction or interruption of enteral nutritional supply is considered separately for the total number of events during the first 14 days of life and the incidence in cases during the first 14 days of life*.

Finally, the observed differences in enteral supply between both groups were analyzed in the context of breast milk supply, milk fortification, and for the clinical item “feeding intolerance.” Rates of breast milk supply (49.3 vs. 47.6%) and milk fortification (72.2 vs. 71.4%) were equally distributed between both groups. The number of cases fulfilling the objective criterion of feeding intolerance on day 1 (22.2 vs. 20.5%) and within the first 14 days of life (97.9 vs. 100%) did not differ between the two groups, but enteral nutrition was paused more frequently in the moderate/severe BPD group (Table 7).

Taken together, the total energy supply during the first 2 weeks of life prevailed as the main criterion separating infants with better and worse pulmonary prognosis, and the detected differences in enteral nutrition were not due to objective criteria of feeding intolerance.

## Discussion

Early and progressive enteral nutritional supply has beneficial effects on somatic growth and psychomotor development. Our results are in line with the sparse literature available on this topic in the context of BPD. Our retrospective analysis demonstrates that even slight differences in early nutritional supply during the first 2 weeks of life can have consequences for the lung. Lower total caloric intake can mainly be attributed to a lower increase in enteral nutrition. Of clinical importance, the overall slower increase in enteral nutrition in the moderate/severe BPD group was not hampered by objectifiable clinical criteria and the prevalence of documented feeding intolerances. Instead, the individual subscribers' subjective assessment of feeding tolerance without a documented objectifiable reason in the patient records was responsible for the differences in enteral nutritional supply. The trend toward increased total fluid intake observed in infants with no/mild BPD is probably due to the fluid volume required to achieve the higher caloric intake. In line with many publications, the immediate period after birth represents an especially vulnerable period for distortion of further lung development that cannot only be assigned to fetal predisposition, infections, mechanical ventilation, and oxygen toxicity (1, 2, 5, 35). The presented datasets extend the rare knowledge on how to optimize nutrition with respect to the pulmonary outcome and enable a clearer view on this important topic (8–10). Adherence to nutrition protocols to reach rapid enteral feeding advances is easily achievable but requires stringency and persistence of each team member.

Our data are not surprising considering the effects of intrauterine growth restriction on the pulmonary outcome. Our data underline that the highly orchestrated process of lung development in the saccular stage needs adequate nutritional supply to meet requirements (1). The comparatively moderate impact of nutrition in our analysis is reflected by the high number of study patients recruited with a resulting *p*-value of p = 0.044. Vice versa, the slight differences in caloric intake of ~10% during the first 2 weeks of life encourage future studies to address the potential of an even more progressive nutritional approach. Fittingly, one recent study on this topic demonstrated the benefit of reaching a higher caloric intake even in week 3 and 4 of life (36). Our data on the lungs are in line with observations that early progressive nutritional supply and the quality of nutrition have a relevant impact on somatic growth and other important outcome parameters (14, 37, 38). Preserved fluid restriction might have the potential to augment the effect size and should be included in the objective of any future study directed to the improvement of the pulmonary outcome (7). High protein intake is of much higher impact on somatic growth and neurodevelopmental outcome compared with carbohydrates and lipids. High protein intake and vitamin A supplementation are also associated with a better pulmonary outcome (9, 16). Due to this well-known huge impact, special focus is directed toward adequate provision during clinical routine for more than a decade. This explains why the preterms in both study groups received identical amounts of protein and vitamin A during the whole study period. Carbohydrates represent one main source of energy. Their importance for growth and lean body mass has been documented recently (36). Our data provide evidence for the importance of carbohydrates for lung development. The results are supported by recent preclinical data that argue for a much more differentiated consideration of nutrition and BPD (39). Future studies will have to address the specific contribution of single nutrients in analogy to the available data for somatic growth and psychomotor development (40–42).

The limitations of our study comprise the retrospective character of the analysis and the establishment of a correlation which does not allow a causal link. We were not able to include volumes of drug administration into our calculations of total fluid intake, as this was not thoroughly documented in the patient records. The long observation period is another potential source of bias. Yet, despite changes in the ventilatory management over time the BPD rate did not decrease as published before (34). Due to the retrospective nature of our study, baseline confounders including growth restriction were considered but it was not possible to retrieve standardized information on, e.g., the PDA status. On the other side, limitations are outweighed by the number of study participants, the low dropout rate due to missing data (0.3%) and risk adjustment for relevant confounders of BPD. Due to the limited number of achievable prospective randomized controlled multicenter trials, the evaluation of the impact of nutritional supply on the incidence and severity of BPD within clinical registries represents a promising alternative. The huge variations in nutritional supply in different centers and the changes in nutritional supply over time represent excellent preconditions for future registries to highlight the impact of optimized nutritional supply not only for somatic growth and psychomotor development but also for the pulmonary outcome.

Besides, the well-established pulmonary risk factors of immaturity, infection, mechanical ventilation, and oxygen toxicity, the contribution of genetic variations, and the microbiome to BPD have come into focus of research during the recent years. We add further data to another dimension of the complexity and the ever-growing list of risk factors for the immature lung. In summary, our data suggest that early high caloric intake achieved by increased supply of carbohydrates is correlated with a better pulmonary outcome in preterm infants. They encourage paying special attention to decipher the ideal nutritional requirements for unrestricted lung development and promoting rapid enteral feeding advances in the absence of objective criteria of feeding intolerance.

## Data Availability Statement

The raw data supporting the conclusions of this article will be made available by the authors, without undue reservation.

## Ethics Statement

The study was conducted following the rules of the Declaration of Helsinki of 1975, revised in 2013. The study was approved by the Ethics Committee of the Justus-Liebig-University Gießen (AZ 98/14). Written informed consent to participate in this study was provided by the participants' legal guardian/next of kin.

## Author Contributions

HE initiated the retrospective analysis. TT, AW, JN, SS, and TL collected the data, performed the validity data checks, and provided valuable intellectual contributions. TT and HE drafted the manuscript and prepared the figures and tables. K-PZ and GE revised the manuscript for important content. All authors have read and approved the final version of the manuscript.

## Funding

This work was supported by von-Behring-Röntgen Stiftung (63-0009) and Clinical Research Unit KFO 309-2. Datasets are deposited in the DZL data warehouse (https://dzl.de/en/dzl-data-warehouse/).

## Conflict of Interest

The authors declare that the research was conducted in the absence of any commercial or financial relationships that could be construed as a potential conflict of interest.

## Publisher's Note

All claims expressed in this article are solely those of the authors and do not necessarily represent those of their affiliated organizations, or those of the publisher, the editors and the reviewers. Any product that may be evaluated in this article, or claim that may be made by its manufacturer, is not guaranteed or endorsed by the publisher.
